# Association of myocardial iron deficiency based on T2* CMR with the risk of mild left ventricular dysfunction in HIV-1-infected patients

**DOI:** 10.3389/fcvm.2023.1132893

**Published:** 2023-04-12

**Authors:** Chengxi Yan, Ruili Li, Jiannan Zhang, Li Zhang, Minglei Yang, Qiujuan Zhang, Hongjun Li

**Affiliations:** ^1^ Department of Radiology, The Second Affiliated Hospital of Xi'an Jiaotong University, Xi'an, China; ^2^ Department of Radiology and Nuclear Medicine, Xuanwu Hospital, Capital Medical University, Beijing, China; ^3^ Department of Radiology, Beijing Youan Hospital, Capital Medical University, Beijing, China; ^4^ Department of Algorithm, Artificial Intelligene Innovation Center (AIIC), Midea Group, Beijing, China

**Keywords:** cardiovascular magnetic resonance, HIV-1, myocardial iron levels, myocardial iron deficiency, left ventricular systolic dysfunction

## Abstract

**Objectives:**

This study sought to noninvasively determine myocardial iron levels in HIV-1-infected patients using CMR and explore the association between T2* values and mild left ventricular systolic dysfunction (LVSD).

**Methods:**

This prospective study was conducted from June 2019 to July 2021. HIV-1-infected adults and healthy controls were consecutively enrolled for CMR exam. CMR exam included the assessment of myocardium iron content (T2*), cardiac function (cine), inflammation (T2), and fibrosis (through extracellular volume fraction [ECV] and late gadolinium enhancement [LGE]) measurements. Mild LVSD is defined as a left ventricular ejection fraction (LVEF) between 40% and 49%.

**Results:**

Of 47 HIV-1-infected patients enrolled, 12 were diagnosed with mild LVSD (HIV-1+/LEVF+) and 35 were diagnosed with preserved LV function (HIV-1+/LEVF−). Compared with healthy controls, HIV-1-infected patients displayed higher T2*, T1, T2, ECV values and lower global circumferential strain (GCS) and global radial strain (GRS) (all *P* < 0.05). However, between patients with and without mild LVSD, only the T2* values and ECV (all *P* <0.05) were different. The association between increased T2* values (>26 ms) and mild LVSD remained significant after adjusting for the established univariate predictors (ECV >32.9%, T1 values >1336 ms) of mild LVSD (odds ratio [OR], 10.153; 95% confidence interval [CI] 1.565–65.878, *P *= 0.015).

**Conclusions:**

Myocardial T2* values were elevated in HIV-1-infected patients, supporting the notion that ID was associated with mild LVSD. Our findings highlight the potential for ID in HIV-1-infected patients as an auxiliary biomarker to monitor the course of LVSD.

## 1. Introduction

Iron deficiency (ID) is believed to be prevalent among HIV-1-infected patients and is associated with a decreased physical capacity and increased mortality (1–3). It is also becoming recognized that iron plays an essential role in cardiovascular disease, especially in chronic heart failure (HF) (4, 5). Notably, ID is also known to drive mitochondrial abnormalities in cardiac muscle (6) by modulating cellular energy functions (7, 8) and regulating mitochondrial biogenesis (9). Thus, correcting ID in HIV-1 patients is necessary to improve anemias and reduce cardiovascular complications (10).

Circulating biomarkers, such as erythrocyte indices, serum iron, ferritin, and soluble transferrin receptor, have been widely used to identify ID in clinical settings because these tests are commonly available and easy to perform. Nevertheless, the accuracy of these tests is often negatively affected by inflammatory states and renal and liver conditions, which are more common in the context of HIV-1 infection (11). However, studies have shown that these circulating biomarkers are poor indicators of myocardial iron levels (12, 13).

With the advancement of magnetic resonance imaging (MRI), T2* cardiac MR (CMR) imaging has become widely used to assess myocardial iron overload in hematologic diseases, such as thalassemia (14). Studies have also shown that changes in iron within the myocardium to be highly correlated with intramyocardial hemorrhage in ischemic heart disease (15). However, only a limited number of studies have reported utilized T2* CMR for ID in non-ischemic cardiomyopathies (16, 17). Notably, the assessment of myocardial iron content in patients using CMR has not been reported in HIV-1-infected patients. We investigated whether myocardial iron content in HIV-1-infected patients is altered based on T2* CMR and whether those changes are associated with systolic dysfunction observed in HIV-1 patients.

## 2. Methods

The institutional ethics committee approved this prospective case-controlled study, and all participants gave written informed consent prior to CMR examinations. The data underlying this article will be shared on reasonable request to the corresponding author.

### 2.1. Study participants

HIV-1-infected patients (*n* = 49) were enrolled in this observational study at Beijing You'an Hospital from June 2019 to July 2021. Inclusion criteria were age ≥18 years and a confirmed HIV-1 diagnosis. Exclusion criteria were history of cardiovascular disease, contraindications for CMR, an estimated glomerular filtration rate of <30 ml/min, and impaired liver function (alanine aminotransferase greater than twice the normal upper limit). Clinical histories, physical examinations, and laboratory data were obtained from the enrolled patients, including a detailed review of the HIV-1 disease stage, antiretroviral therapy (ART) exposure, and cardiovascular disease risk factors. In the same patients, fasting lipid panels, glucose levels, creatine levels, serum ferritin (SF) and current plasma CD4^+^ T-cell counts, CD4^+^/CD8^+^ ratios, HIV-1 loads, and hematocrit levels were also acquired. Mild LVSD was defined as an LVEF measured between 40% and 49% (18). Anemia was defined as hemoglobin <13 g/dl in men and <12 g/dl in women (19). The acquired immunodeficiency syndrome (AIDS) stage was defined as the symptomatic stage when the virus becomes highly active and the immune system of patient weakens as reported previously (20). The control group consisted of an age- and ethnicity matched (self-defined) subjects (*n* = 21) with no history of HIV-1 infection or cardiovascular disease. CMR studies and subsequent analyses were performed in a blinded manner, with sequential numbering of subjects. Detailed inclusion and exclusion criteria for participants and healthy control participants were shown in previous study (21).

### 2.2. Cardiac magnetic resonance acquisition and image analysis

Details of the imaging sequences and image analysis are provided in the supplementary data online with chronological sequence of the applied CMR techniques reported in Supplementary Figure S1. CMR was performed for patients with 3.0-T systems (MAGNETOM Trio, Siemens Medical Systems, Erlangen, Germany). T2* imaging was performed to assess myocardial iron content; cardiac function was evaluated by steady-state free precession cine images. Motion-corrected myocardial relaxation maps (T1, T2) were used to estimate the mean T1 and T2 values. Hematocrit-corrected ECV values were determined using native and post-contrast T1 values. LGE images were evaluated qualitatively for the presence or absence of enhancements.

### 2.3. Inter- and intra- observer variability

Intra- and inter-observer variabilities for the CMR parameters were assessed in 40 randomly selected participants (including patients and healthy controls). To evaluate the intra-observer variability, the selected images were blindly reanalyzed by the same observer after a “washout” period of 2 weeks. To evaluate inter-observer variability, all selected images were presented in random order to two readers, who were blinded to patient details and the other reader's findings.

## 3. Statistical analysis

All statistical analyses were performed using SPSS (version 23.0, IBM statistics, Armonk, NY, USA) and GraphPad Prism software (Version 8.1, GraphPad Software Inc). Continuous variables were expressed as mean ± SD or median ± interquartile range depending on the normality of the data. Categorical variables were expressed as counts (percentages). The Shapiro-Wilk test was used to test if the data were distributed normally. Categorical variables were compared using the c^2^ test (with a cell count >5) or the Fisher's exact test (with a cell count ≤5). Continuous variables were compared among the groups using a one-way analysis of variance or independent t-test analysis (for normal distributions) and Kruskal-Wallis tests (for non-normal distributions). Post-hoc analysis was performed for pairwise group comparisons. The Spearman rank correlation test was used to test correlations between the variables. Quantitative variables were transformed into categorical variables according to their normal range or cutoff values provided by receiver operating characteristic (ROC) curve analyses. A univariable and multivariable binary logistic regression analysis was applied to test the impact of clinical and CMR imaging variables on being able to predict mild LVSD in the 41 patients who underwent CMR examinations with Gd injection. After a forward selection of relevant covariates with a *P *< 0.1 for the univariable analysis, covariates were added to a multivariable model to assess the impact of the variables further. Reproducibility was assessed using a Bland-Altman analysis, linear regression, and intraclass correlation coefficients. The level of statistical significance was set at *P *< 0.05.

## 4. Results

### 4.1. Participant characteristics

Among the 49 HIV-1-infected patients recruited, two patients were excluded because of poor-image quality. In the remaining 47 patients, 41 patients underwent CMR with Gd injections, and 6 patients refused Gd injections. HIV-1-infected patients were divided into two groups, those with HIV-1 and mild LVSD (HIV-1+/LVEF+, *n* = 12) and those with HIV-1 and preserved LV function (HIV-1+/LVEF−, *n *= 35). None of the subjects showed evidence for LVEF <40%. In addition, 21 normal control subjects were included in the final analyses, in all of whom images were of diagnostic quality, and every subject underwent Gd-enhanced CMR. The demographic and clinical characteristics of the study population are summarized in Supplementary data online, Supplementary Table S1. In keeping with previous reports, where the ratio of reported infections was four-folder greater in men than in women (22), more men were found in HIV-1 infection group compared with healthy control subjects (*P* < 0.001). Furthermore, patients with and without mild LVSD had a higher occurrence of hypertension than the control subjects.

### 4.2. CMR tissue characterization—T2*, T1, T2, ECV and LGE

**Figure 1:**
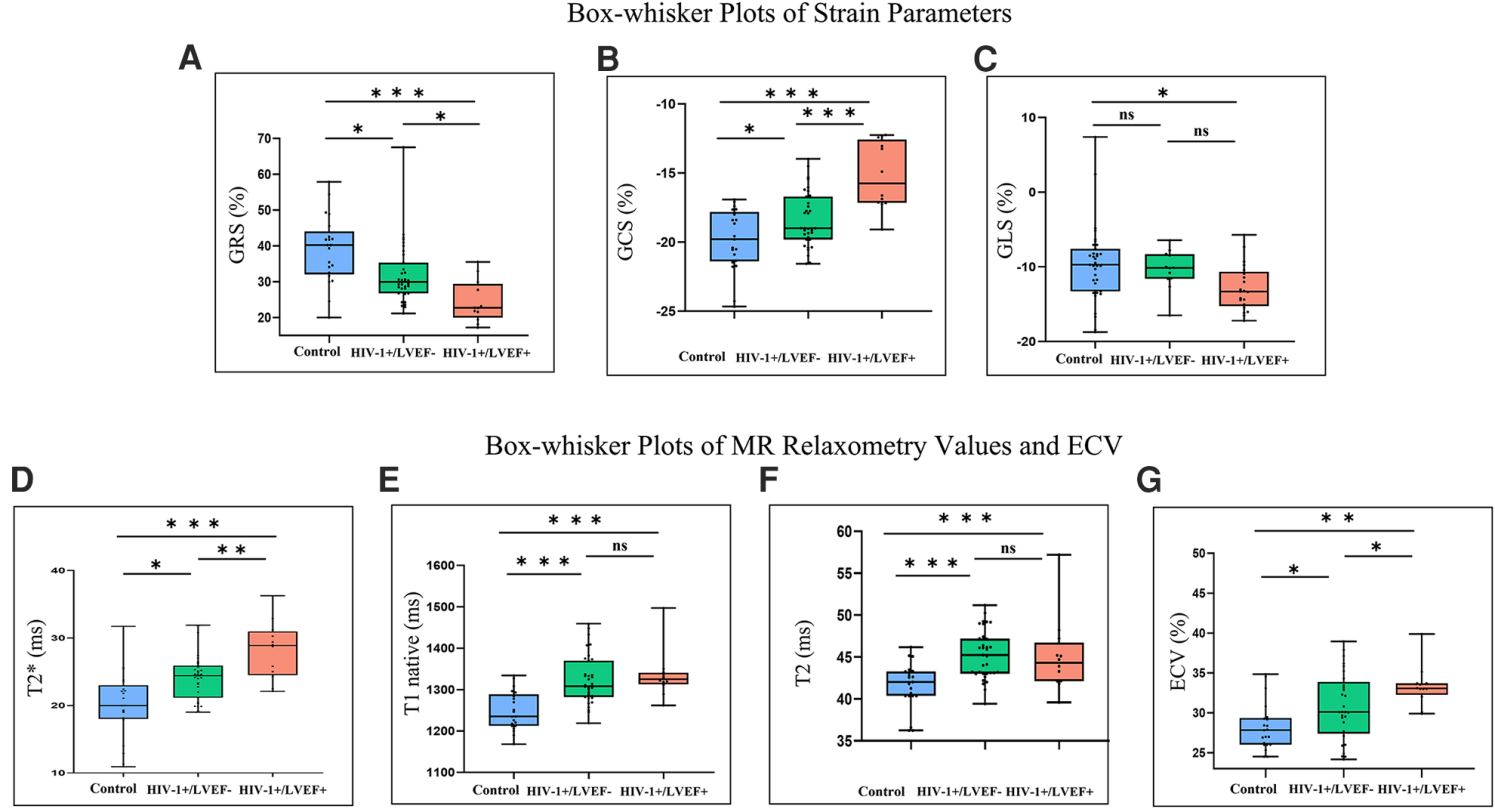
Box-whisker plots of cardiac magnetic resonance (CMR) parameters determined from control subjects and HIV-1-1infected patients with preserved LV function (HIV-1+/LVEF-) and those with LV dysfunction (HIV-1+/LVEF+). The top and bottom horizontal lines represent the maximum and minimum of the data, respectively. The colored boxes represent the data between the first and third quartiles. The horizontal lines in the middle of the colored boxes represent the median. Differences are shown for GRS **(A)**, GCS **(B)**, GLS **(C)**, T2* values **(D)**, T1 native **(E)**, T2 **(F)** and ECV **(G)**. * Indicates significant pairwise comparison (*P* < 0.05), ** indicates significant pairwise comparison (*P* < 0.01), *** indicates significant pairwise comparison (*P* < 0.001). GRS, global radial strain; GCS, global radial strain; GLS, global longitudinal strain; ECV, extracellular volume; LGE, late gadolinium enhancement.

**Figure 2:**
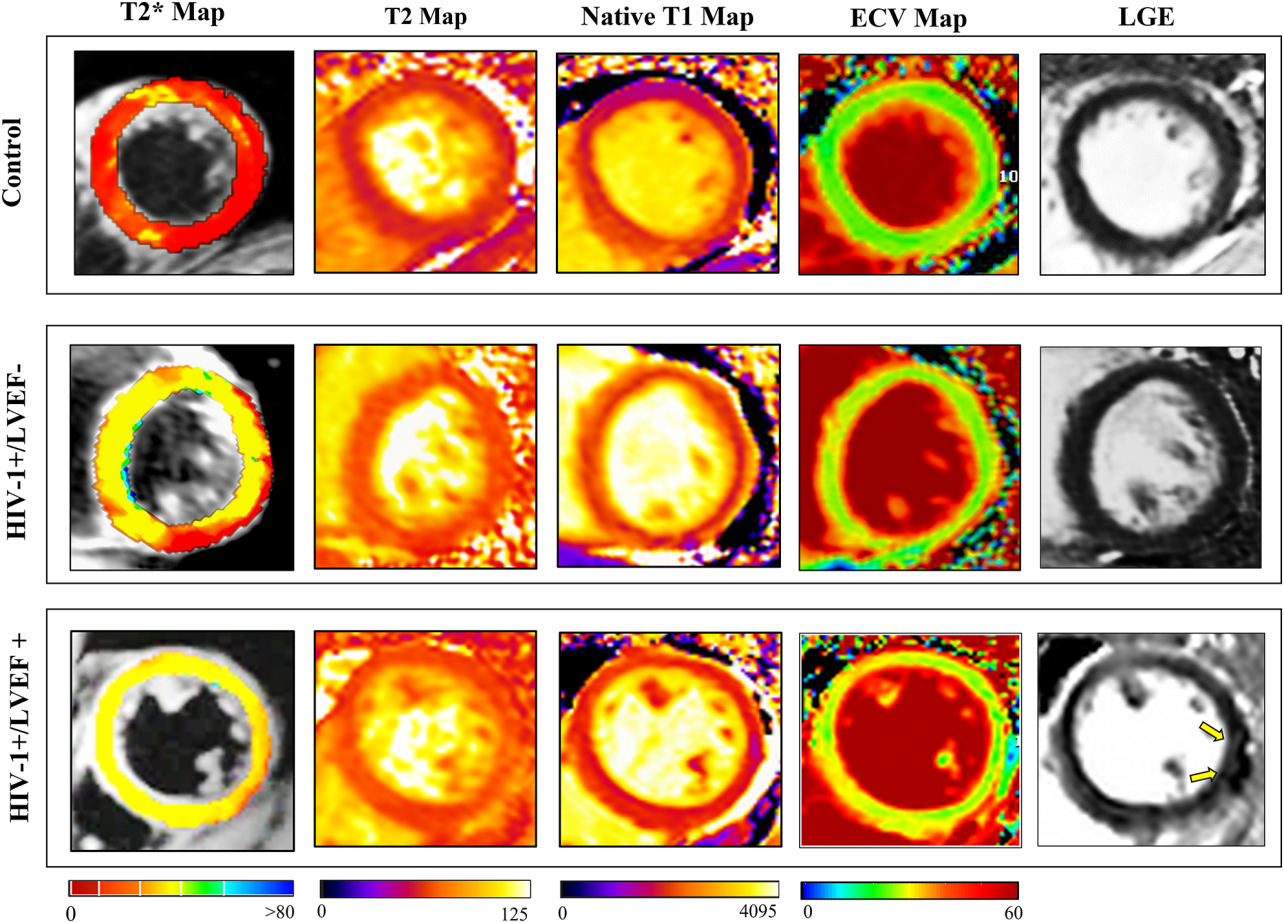
Representative clinical examples of CMR images (T2*, T2, T1 native, ECV and LGE) obtained from a healthy subject (control, 35-year-old male), LVEF = 60.3% (top row), a HIV-infected patient (30-year-old male, with preserved LVEF (55.6% (middle row)), and a HIV-infected patient (34-year-old woman with LV dysfunction (LVEF = 45.1%) (bottom row). Quantitative parameters are considerably higher in HIV-infected patients and increase proportionately with increasing severity of left ventricular ejection fraction (LVEF), LGE showed patchy enhancement in HIV-1+/LVEF+ patient (yellow arrows). Abbreviations as in Figure 1.

**Figure 3:**
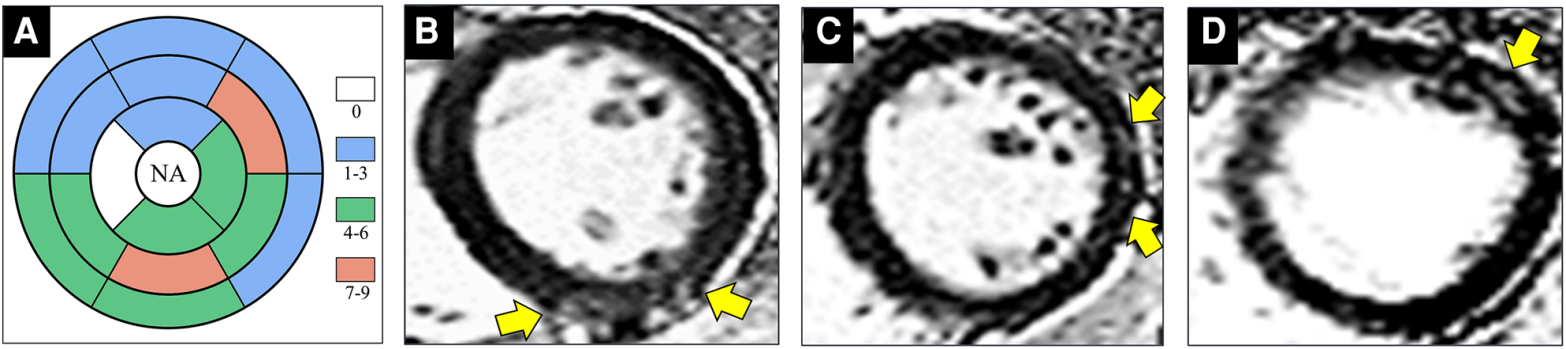
Images show representative examples of HIV-1-infected patients with positive late gadolinium enhancement (LGE). **(A)** Distribution of positive LGE according to segments of myocardium indicates preferable involvement of mid- subepicardial-wall in the inferior segments and superior-lateral segments. **(B)** 30-year-old male patient underwent cardiac magnetic resonance (CMR) imaging after one year of HIV diagnosis. Phase-sensitive inversion recovery (PSIR) sequences in short-axis view shows focal enhancement (yellow arrow) at mid-inferior segment. **(C)** A 34-year-old male patient underwent CMR after one year of HIV diagnosis. Sub-epicardium enhancement (yellow arrow) is visible at mid-inferolateral segment in a patchy pattern. **(D)** A 29-year-old male patient underwent CMR after five years of HIV diagnosis. Short-axis PSIR image reveals sub-epicardial enhancement at mid-anterolateral segment (yellow arrow). NA = not apply. Abbreviations as in Figure 1.

Group comparisons of myocardial T2* values are shown in Table 1, Figure 1. T2* values were significantly lower in controls than HIV-1+/LVEF+ (21.1 ± 5.0 ms vs. 28.6 ± 3.8 ms, *P* < 0.001) and HIV-1+/LVEF− (21.1 ± 5.0 ms vs. 24.3 ± 3.1 ms, *P* < 0.05). Native T1 values, T2 values, and ECVs were also significantly different among the three groups. Compared with HIV-1+/LVEF−, HIV-1+/LVEF+ had higher ECVs (Table 1, Figure 2). LGE was positive in 10 out 41 HIV-1-infected patients but none of the normal controls were positive. There was no difference in the proportion of LGE between HIV-1+/LVEF− vs. HIV-1+/LVEF+ (*P* > 0.05). Patchy LGE was preferably located at mid- subepicardial-wall in the inferior segments and superior-lateral segments (Figure 3).

**Table 1:** Cardiac magnetic resonance imaging findings.

	HIV-1+ /LVEF+ (*n* = 12)	HIV-1+ /LVEF− (*n* = 35)	Normal Controls (*n* = 21)	*P* value
**Cardiac MRI parameters**				
LVEF (%)	46.7 ± 2.3^a,b^	58.8 ± 4.7^a^	62.8 ± 8.1	<0.001*
GRS (%)	24.2 ± 5.9^a,b^	31.9 ± 8.5^a^	38.9 ± 9.4	<0.001*
GCS (%)	−15.2 ± 2.4^a,b^	−18.4 ± 1.9^a^	−19.9 ± 2.2	<0.001*
GLS (%)	−9.7 ± 5.1^a^	−10.4 ± 2.6	−12.8 ± 3.1	0.027*
T2* values (ms)	28.6 ± 3.8^a,b^	24.3 ± 3.1^a^	21.1 ± 5.0	0.001*
T2 values (ms)	45.1 ± 4.5^a^	45.3 ± 2.9^a^	41.6 ± 2.7	<0.001*
T1 native (ms)	1322.4 ± 63.7^a^	1292.8 ± 58.6^a^	1248.2 ± 46.5	<0.001*
ECV (%)	32.5 ± 3.1^a,b^	31.5 ± 4.9^a†^	28.2 ± 2.9	0.012*
Visual LGE	3 (25.0)^a^	7/29 (24.1)^a†^	0 (0.0)	0.023*
pericardial effusion	4 (33.3)	5 (14.3)	4 (19.0)	0.290

Data are summarized as the mean ± SD, if they were Gaussian distributed, or the median and interquartile range, if they were non-Gaussian distributed, and an *n* (%) was used for categorical variables. The patient denominators included in the analyses are provided if they differed from the group's overall numbers. *P*-values were obtained using one-way ANOVA, Student's *t*-test, Kruskal-Wallis test (for non-normal data), c^2^ test or Fisher's exact test.

Acronyms: LVEF, left ventricular ejection fraction; GRS, global radial strain; GCS, global radial strain; GLS, global longitudinal strain; ECV, extracellular volume; LGE, late gadolinium enhancement.

^a^ *P* < 0.05 vs. normal control subjects,.

^b^ *P* < 0.05 vs. HIV with preserved LV function,.

* Indicates a significant difference.

^†^ Indicates *n* = 29.

### 4.3. Strain values

**Figure 4:**
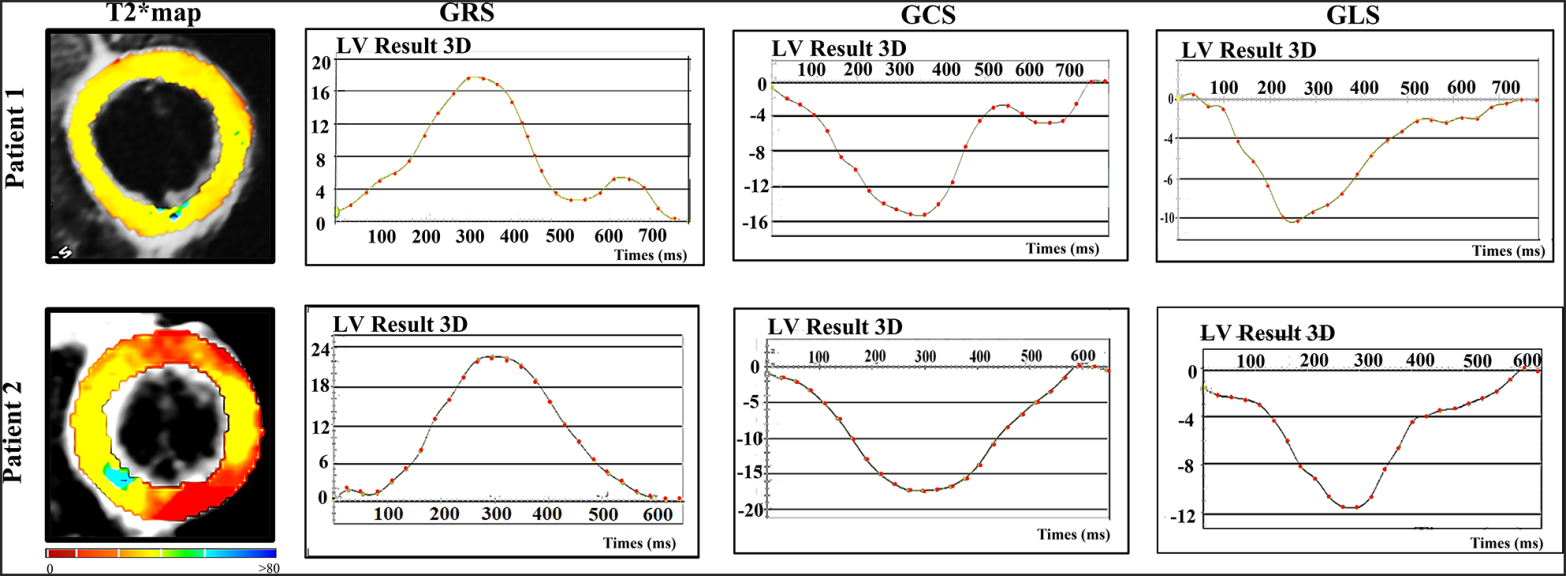
Representative examples of T2* maps and corresponding strain patterns (GRS, GCS and GLS) in two patients. Patient 1 - 38 years-old asymptomatic male with HIV diagnosis of 10 years; Patient 2 - 37 years-old male asymptomatic male with HIV diagnosis of 2 years. Abbreviations as in Figure 1.

Mean global radial, circumferential and longitudinal strains showed increasing impairment from the control, preserved LVEF, to mild LVSD groups (all *P* < 0.05). However, only the global radial and circumferential strains were different between HIV-1+/LVEF+ and HIV-1+/LVEF− groups. See Figures 1, 4, Table 1.

### 4.4. Associations among clinical variables, CMR characteristics, and HIV-1 infected patients

T2* values, ECV, GRS, and GCS values were correlated with mild LVSD in HIV-1-infected Patients (all *P* < 0.05, Table 2). For the logistic regression analysis, quantitative variables were transformed into categorical variables according to their normal values and cutoff values provided by ROC analysis. Cutoff values for the T2*, T2, native T1 values, and ECVs were 26 ms, 47 ms, and 1336 ms, and 32.9% respectively. Univariable binary logistic regression analyses revealed an association between mild LVSD and T2* values (odds ratio [OR]: 9.667; 95% confidence interval [CI] 2.184–47.663; *P *= 0.003), native T1 values (OR: 5.294; 95% CI 1.010–27.748; *P *= 0.049), and ECVs (OR: 9.583; 95%CI 1.802–50.956; *P *= 0.008) (at *P *= 0.1). In the multivariable binary logistic regression model, T2* values were significantly associated with mild LVSD (OR: 10.153; 95% CI 1.565–65.878; *P *= 0.015) (Table 3).

**Table 2:** Relationship between clinical and CMR parameters and LV dysfunction in all HIV-1 patients.

	*r* Value	*P* Value
**Variables**		
Hypertension	0.107	0.384
SF (ng/ml)	0.081	0.317
Hemoglobin	−0.104	0.071
GRS	−0.450	0.002*
GCS	0.518	<0.001*
GLS	0.350	0.056
T2* values (ms)	0.472	0.001*
T2 values (ms)	0.112	0.445
T1 native (ms)	0.142	0.341
ECV (%)	0.313	0.047*
Visual LGE	0.216	0.176

SF, serum ferritin. Other abbreviations as in Table 1.

* Denotes significant values.

**Table 3:** Multivariable model of LV dysfunction with T2* adjusted to univariate clinical and CMR predictors (at *P* < 0.1).

	Univariate OR (95% CI)	*P* value	Multivariate OR (95% CI)	*P* value
**Variables**				
Hypertension	2.008 (0.465, 8.597)	0.352	–	–
SF (ng/ml)	0.891 (0.718, 1.316)	0.214	–	–
Hemoglobin	4.513 (0.981, 8.784)	0.061	–	–
T2* values (ms)	9.667 (2.184, 42.793)	0.003	10.153 (1.565, 65.878)	0.015*
T2 values (ms)	0.976 (0.797, 3.128)	0.547	–	–
T1 native (ms)	5.294 (1.010, 27.748)	0.049	2.354 (0.321, 17.261)	0.400
ECV (%)	9.583 (1.802, 50.956)	0.008	12.166 (1.653, 89.534)	0.014*
Visual LGE	4.286 (0.764, 38.638)	0.195	–	–

* Denotes significant values.

Abbreviations as in Tables 1, 2.

### 4.5. Inter- and intra- observer variability

**Figure 5:**
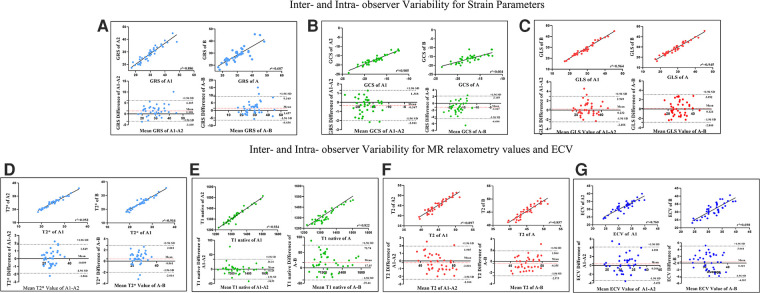
Linear Regression and Bland-Altman analyses for GRS **(A)**, GCS **(B)**, GLS **(C)**, T2* values **(D)**, T1 native **(E)**, T2 values **(F)** and ECV values **(G)** for inter-observer and intra-observer variabilities. The solid line represents bias. The horizontal red dashed line depicts the mean; the two black dashed lines depict the upper and lower limits of agreement (+ 1.96 SD and − 1.96, respectively). A1 The initial measurement of the first observer, A2 one month after the initial measurement of the first observer, A Mean value of A1-A2, B The second observer. Abbreviations as in Figure 1.

For strain parameters, MR relaxometry values and ECV inter-observer variability showed close agreement between readers (ICC >0.93). Similarly intra-observer variability for the same parameters was also in agreement (ICC >0.82). Refer to Table 4, Figure 5 for details.

**Table 4:** Reproducibility of CMR parameters.

	Intra-observer			Inter-observer		
	Bland-Altman analysis			Bland-Altman analysis		
	Bias	95% Limits of Agreement	ICC	Bias	95% Limits of Agreement	ICC
**Variables**						
GRS	1.398	−3.410 to 6.205	0.967	1.457	−6.436 to 9.349	0.894
GCS	−0.347	−2.011 to 1.318	0.967	−1.167	−4.494 to 2.160	0.828
GLS	0.232	−2.486 to 2.949	0.977	0.126	−2.840 to 3.092	0.972
T2* values (ms)	−0.218	−2.465 to 2.030	0.974	1.233	−2.014 to 2.818	0.954
T1 native (ms)	0.125	−39.44 to 73.74	0.966	17.15	−39.44 to 73.14	0.879
T2 values (ms)	−0.231	−2.366 to 1.905	0.939	−0.355	−2.575 to 1.864	0.911
ECV (%)	0.356	−3.452 to 4.181	0.931	0.369	−4.305 to 5.044	0.907

Abbreviations as in Table 1.

## 5. Discussion

In a univariate model, our study revealed that the myocardial T2* was independently associated with mild LV dysfunction in HIV-1 infected patients. This association remained significant even after adjusting for other imaging co-variates in a multivariate model. Importantly, we found that the T2* of myocardium of HIV-1 infected patients to be significantly elevated compared to matched controls, suggesting myocardial iron deficiency is a hallmark in HIV-1 patients. Our findings here also lend early support for the hypothesis that restoration of iron could be a potential strategy to mitigate the functional impairment in HIV-1 infected patients.

Although systemic ID has been previously described, the present study is the first to demonstrate myocardial iron deficiency in HIV-1 subjects (23). Reduced myocardial iron level might result from systemic iron derangements due to decreased iron intake, poor bioavailability, and repeated HIV-1 infections (24). Also, higher level of hepcidin in both treated and untreated HIV patients can inhibit iron release by macrophages, decrease iron absorption through the internalization and impede ferroproteins degradation in enterocytes and macrophages (25). From our data, it is not possible to determine whether myocardial iron deficiency in HIV-1 infected patients were resulted from relative iron deficiency (decreased systemic iron availability despite overall normal total/body iron) or absolute iron deficiency (reduced iron stores). There was no significant difference among HIV+/LVEF+, HIV+/LVEF−, and healthy controls in hemoglobin and serum ferritin (Supplementary Table S1). Also, there was no correlation between T2* values and those indexes. Besides, none of the patients in this study had decreased SF despite a significant proportion of patients (9/47, 19.1%) were identified to have anemia (10). These facts may reflected a lack of agreement between traditional markers of ID and myocardial tissue iron, as shown in previous publications (26). Previous direct tissue analyses demonstrated that myocardial iron content might be independent of absolute iron regulation (27). In addition, as an acute reactant that rises with the chronic inflammation, ferritin is often elevated in HIV-infected individuals regardless of iron status. Thus, Iron stores likely overestimated in HIV-infected populations (28).

Our findings of cardiac dysfunction in the setting of compromised iron are supported by other studies as well. In an *in vitro* human cardiomyocytes experiment, when human embryonic stem cell-derived cardiomyocytes were deprived of iron, cardiomyocyte function was reduced as characterized by impaired mitochondrial respiration and reduced contractility and relaxation (29). In other studies, on the basis of T2* CMR (16, 17, 30–32), elevated myocardial T2* values (hence reduced iron content) has been shown to occur in non-ischemic heart failure (16, 32), hypertrophic cardiomyopathy (31), and hemodialysis-dependent end-stage renal disease (ESRD) (17, 30). Increased T2* has also been shown to be a an independent risk factor for LV dysfunction in ESRD patients (17) and a predictor of adverse cardiac events in non-ischemic heart failure (16).

Although the range of T2* values in the healthy controls of our study was lower than that reported by Meloniet al. (33), our findings are close to Xu a (17). Besides, the T2* values in HIV-1+/LVEF− were similar to those with non-ischemic cardiomyopathy (16). Even the extent of myocardial iron deficiency we observed in HIV-1+/LVEF+ did not reach the levels reported in patients with ESRD (17, 30) as it was considerably higher than the HIV-1+/LVEF− patients and normal control subjects of our study. Due to a large fraction of asymptomatic patients (63.8%, 30 of 47) enrolled in our study, the iron metabolism disorder might have been relatively mild compared with the patients in end stage of renal disease. Additional studies with larger sample sizes are needed to explore whether the myocardial iron deficiency in patients the late stages of HIV-1 disease also experience more severe impairment in LV function.

Furthermore, we found that CMR markers of myocardial inflammation were elevated in HIV-1-infected patients, indicating subclinical myocardial inflammation. Previous CMR studies revealed that HIV-1-related cardiovascular disease is associated with chronic inflammation, frequent pericardial effusion, and probable myocardial edema (33–35). Increases in T2 and native T1 relaxation times, as a quantitative CMR measure of water content, although nonspecific, can be seen in myocardial infarction, myocarditis, amyloidosis, and focal and diffuse fibrosis (36–38). It is worth noting that we observed higher ECV in HIV-1+/LVEF+ than HIV-1+/LVEF− and normal controls, which has also been designated a risk factor for mild LV dysfunction. Increased ECV values were found to be associated with the severity of diffuse myocardial fibrosis as identified on histopathology. ECV values were also a major determinant of altered diastolic filling and LV systolic pumping function (39). Studies have also shown a correlation between ECV and LV function in patients with end-stage HF secondary to mitral regurgitation (MR) (40). We note that the range of ECV values in the healthy controls of our study were higher than previously reported by Julian et al. (33), they were close to a Chinese cohort in a study by Lin et al. (41).

It is possible that T2* values may also be influenced by edema, but it is far less insensitive compared with T2-based methods (15, 42). On 1.5 T MRI, in patients with acute non-hemorrhagic myocardial infarction experienced an increase in T2* of 6%, but 78% increase in T2-weighted signal intensity with respect to remote myocardium (42). Similar findings have been found in large animals' models as well with more than a 3-fold greater difference in the sensitivity to edema in T2-based vs. T2*-based approaches (15). These observations along with our observations in T2 and T2* in HIV-1 patients here suggest that the increase in T2* in HIV-1 patients we observed is likely a consequence of ID than from edema. Besides, our previous study (21) had been reported a higher rate of subclinical myocardial edema (represented by T2 values) and fibrosis (represented by LGE) in HIV-infected patients, but we further found that it was fibrosis but not edema that changed with the severity of the disease. In this study, we also found that HIV patients had higher T2 values than healthy controls, meanwhile, there was no differences in T2 values between patients with normal LVEF and patients with abnormal LVEF. Contrarily, T2* values showed a significantly negative ecoefficiency with LVEF in HIV-infected patients. Thus, we suspected that with the extensively use of antiretroviral therapy, it was chronic inflammation rather than acute inflammation that existed in HIV patients, and edema alone cannot explain the increasing in T2* values between patients with and without normal LVEF.

## 6. Limitations

There are several limitations in this study. First, myocardial T2* measurement in clinical practice is typically performed at 1.5 T, and our studies are performed at 3.0 T, where B0 and B1inhomogeneities are greater (43). Despite this we found our data to be reproducible. Further, the use of 3.0 T for CMR exams is growing rapidly given the well-known SNR benefits that can be exchanged for imaging speeds and higher-spatial resolution. Next, none of the HIV-1 patients in our study had severe LV dysfunction. Thus, additional studies in symptomatic patients are needed to evaluate whether iron deficiency and greater impairment in LV dysfunction are linked. On the other hand, iron overload has been shown to be associated with increased susceptibility to certain infections (44). Therefore, prophylactic iron supplementation without repeat assessment for myocardial iron can facilitate the development of opportunistic infections. Hence, additional studies are required to establish the benefits of using T2* CMR for guiding appropriate iron treatment strategies. Further, our findings are determined from a cross-sectional and exploratory study with a scant number of patients, so it was impossible to assign causal links between myocardial iron deficiency and altered LV function seen in the HIV-1 group. Longitudinal studies with a larger cohort are warranted to confirm our findings. ART is a potential confounder in the present study. However, with a push toward early HIV-1 treatment, it is unlikely that a large cohort of ART-naive patients would be available for comparative studies to help determine the influence of ART on myocardial iron deficiency. Furthermore, given the diverse combinations of antiretroviral drugs used on patients in the present study, we could not identify drug- or drug class-specific effects. This would necessitate a suitably powered prospective study.

## 7. Conclusion

In the present study, we demonstrated a previously underappreciated high burden of iron deficiency in HIV-1 subjects using T2* CMR. We found that myocardial iron deficiency is an independent risk factor and predictor of LV dysfunction. Further studies are required to determine whether changes in iron metabolism plays a causal role in the increased cardiac morbidity and mortality of HIV-1-infected patients and whether iron supplementation could improve the management of HIV patients.

## Data Availability

The original contributions presented in the study are included in the article/**Supplementary Material**, further inquiries can be directed to the corresponding author/s.
