# Supplementary material for: Association of myocardial iron deficiency based on T2* CMR with the risk of mild left ventricular dysfunction in HIV-1-infected patients
Source: Front Cardiovasc Med. 2023 Apr 12;10:1132893. doi: 10.3389/fcvm.2023.1132893 (PMC10130653; doi:10.3389/fcvm.2023.1132893)

Supplementary Material

**Association of Myocardial Iron Deficiency based on T2* CMR with the Risk of Mild Left Ventricular Dysfunction in HIV-1-Infected Patients**

**Chengxi Yan†, MD, PhD1; Ruili Li†, MD, PhD2; Minglei Yang, PhD3; Qiujuan Zhang, MD, PhD1*; Hongjun Li, MD, PhD2***

*** Correspondence:** Dr. Hongjun Li and Dr. Qiujuan Zhang contributed equally to this manuscript and are co-corresponding authors.

Correspondence to Hongjun Li, MD, PhD: Email: lihongjun00113@sina.com

Correspondence to Professor Qiujuan Zhang, MD, PhD: E-mail: [zhangqjlcx@aliyun.com](mailto:zhangqjlcx@aliyun.com)

**Details of the Imaging Sequences and Image Analysis**

CMR was performed for patients with 3.0-T systems (MAGNETOM Trio, Siemens Medical Systems, Erlangen, Germany). Myocardial T2* mapping were obtained using a black blood eight-echo gradient-echo sequence (echo time range 2.7 ms to 18.8 ms; ΔTE 2.30 ms; TR 2 heartbeats; slice thickness 8 mm; field of view 285 mm × 380 mm; matrix 192 × 256 pixels; flip angle: 18^o^. ECG-gated steady-state free precession cine images were obtained. Imaging parameters were: TR/ TE 3.4ms/1.5ms, field of view (FOV) 276 × 340mm^2^, matrix 216× 256, slice thickness 6 mm. T2 mapping was performed using three SSFP images each with different T2 preparation time (TE T2P = 0 ms, 30 ms, 55 ms) were acquired in end-diastole within one breath hold before injection with a contrast agent. Imaging parameters were: TR 219.14 ms; FOV 288×360 mm; matrix 154 × 192; slice thickness 8 mm; flip angle: 48^o^. T1 mapping was acquired using an ECG-gated single-shot modified Look-Locker inversion-recovery (MOLLI) sequence with protocols 5(3)3 and 4(1)3(1)2, respectively, before and 20 min after administering a single bolus of Gadopentetate dimeglumine (Gd; 0.2 mmol/kg body weight), Berlex; Bayer Healthcare, New Jersey. Imaging parameters were: TR/TE 2.7ms/1.1ms; FOV 288×360 mm; matrix 218×256; slice thickness 8mm; flip angle: 35^o^. Late gadolinium enhancement (LGE) imaging based on 2D phase-sensitive inversion-recovery (PSIR) gradient-echo pulse sequence with breath-hold was performed 10 min after contrast administration (short-axis, two chamber, four chamber views). Imaging parameters were: TR/TE 3.4ms/1.6ms; FOV 295×350 mm matrix 295×350; slice thickness 10mm; flip angle: 20^o^.

Two readers with 2 (Y.C.X.) and 16 years (G.X.J.) of CMR experience analyzed the data and performed the measurements in consensus using a commercially available software CVI42 (Version 5.11.2 Circle Cardiovascular Imaging, Calgary, Canada). Midventricular septal segments (8 and 9 as per AHA recommendation21) were used to assess T2* values. LVEF was measured by contouring the endocardium and epicardium on short-axis cine images at the end-systolic and end-diastolic phases. CMR feature tracking was performed using the short-axis, 4-chamber and 2-chamber steady-state free precession cine images. Global systolic radial (GRS), circumferential (GCS), and longitudinal strain (GLS) values were calculated from the peak segmental data. Motion-corrected myocardial relaxation maps (T1, T2) were used to estimate the mean T1 and T2 values. Hematocrit-corrected ECV values were determined using native and post-contrast T1 values. LGE images were evaluated qualitatively for the presence or absence of enhancements, and the location (16 segments of AHA21), and pattern (subepicardial, subendocardial, mid-wall and transmural) of LGE lesions on the LGE images.

**Figure S1: CMR Imaging Protocol**


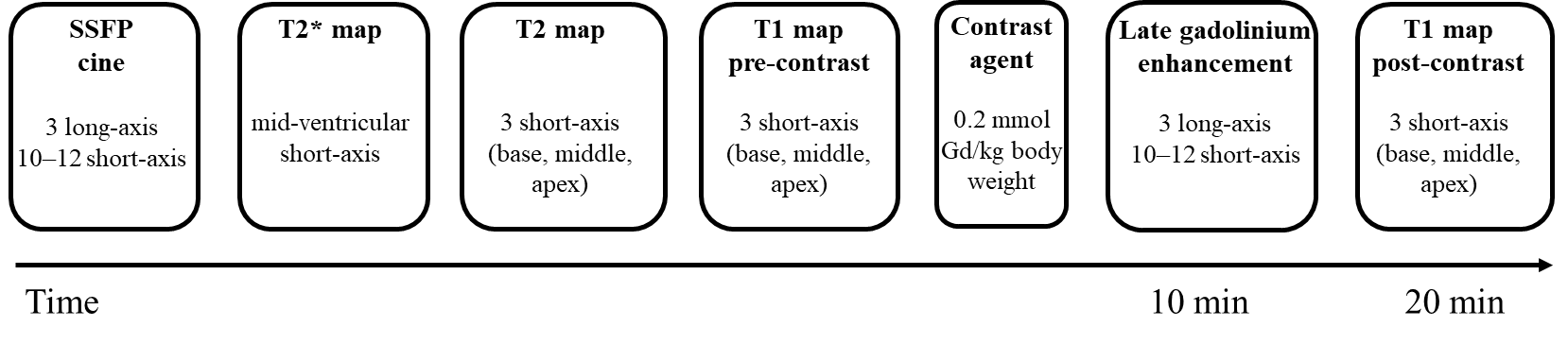

Supplement: Supplementary file 1 [file Table1.docx]
