# Supplementary material for: Association of myocardial iron deficiency based on T2* CMR with the risk of mild left ventricular dysfunction in HIV-1-infected patients
Source: Front Cardiovasc Med. 2023 Apr 12;10:1132893. doi: 10.3389/fcvm.2023.1132893 (PMC10130653; doi:10.3389/fcvm.2023.1132893)
Supplement: Supplementary file 2 [file Table2.docx]

Supplementary Material

**Association of Myocardial Iron Deficiency based on T2* CMR with the Risk of Mild Left Ventricular Dysfunction in HIV-1-Infected Patients**

**Chengxi Yan†, MD, PhD^1^; Ruili Li†, MD, PhD^2,3^; Minglei Yang, PhD^4^; Qiujuan Zhang, MD, PhD^1^*; Hongjun Li, MD, PhD^2^***

*** Correspondence:** Dr. Hongjun Li and Dr. Qiujuan Zhang contributed equally to this manuscript and are co-corresponding authors.

Correspondence to Hongjun Li, MD, PhD: Email: lihongjun00113@sina.com

Correspondence to Professor Qiujuan Zhang, MD, PhD: E-mail: [zhangqjlcx@aliyun.com](mailto:zhangqjlcx@aliyun.com)

**Details of the Imaging Sequences and Image Analysis**

CMR was performed for patients with 3.0-T systems (MAGNETOM Trio, Siemens Medical Systems, Erlangen, Germany). Myocardial T2* mapping were obtained using a black blood eight-echo gradient-echo sequence (echo time range 2.7 ms to 18.8 ms; ΔTE 2.30 ms; TR 2 heartbeats; slice thickness 8 mm; field of view 285 mm × 380 mm; matrix 192 × 256 pixels; flip angle: 18^o^. ECG-gated steady-state free precession cine images were obtained. Imaging parameters were: TR/ TE 3.4ms/1.5ms, field of view (FOV) 276 × 340mm^2^, matrix 216× 256, slice thickness 6 mm. T2 mapping was performed using three SSFP images each with different T2 preparation time (TE T2P = 0 ms, 30 ms, 55 ms) were acquired in end-diastole within one breath hold before injection with a contrast agent. Imaging parameters were: TR 219.14 ms; FOV 288×360 mm; matrix 154 × 192; slice thickness 8 mm; flip angle: 48^o^. T1 mapping was acquired using an ECG-gated single-shot modified Look-Locker inversion-recovery (MOLLI) sequence with protocols 5(3)3 and 4(1)3(1)2, respectively, before and 20 min after administering a single bolus of Gadopentetate dimeglumine (Gd; 0.2 mmol/kg body weight), Berlex; Bayer Healthcare, New Jersey. Imaging parameters were: TR/TE 2.7ms/1.1ms; FOV 288×360 mm; matrix 218×256; slice thickness 8mm; flip angle: 35^o^. Late gadolinium enhancement (LGE) imaging based on 2D phase-sensitive inversion-recovery (PSIR) gradient-echo pulse sequence with breath-hold was performed 10 min after contrast administration (short-axis, two chamber, four chamber views). Imaging parameters were: TR/TE 3.4ms/1.6ms; FOV 295×350 mm matrix 295×350; slice thickness 10mm; flip angle: 20^o^.

Two readers with 2 (Y.C.X.) and 16 years (G.X.J.) of CMR experience analyzed the data and performed the measurements in consensus using a commercially available software CVI42 (Version 5.11.2 Circle Cardiovascular Imaging, Calgary, Canada). Midventricular septal segments (8 and 9 as per AHA recommendation21) were used to assess T2* values. LVEF was measured by contouring the endocardium and epicardium on short-axis cine images at the end-systolic and end-diastolic phases. CMR feature tracking was performed using the short-axis, 4-chamber and 2-chamber steady-state free precession cine images. Global systolic radial (GRS), circumferential (GCS), and longitudinal strain (GLS) values were calculated from the peak segmental data. Motion-corrected myocardial relaxation maps (T1, T2) were used to estimate the mean T1 and T2 values. Hematocrit-corrected ECV values were determined using native and post-contrast T1 values. LGE images were evaluated qualitatively for the presence or absence of enhancements, and the location (16 segments of AHA21), and pattern (subepicardial, subendocardial, mid-wall and transmural) of LGE lesions on the LGE images.

**Figure S1: CMR Imaging Protocol**


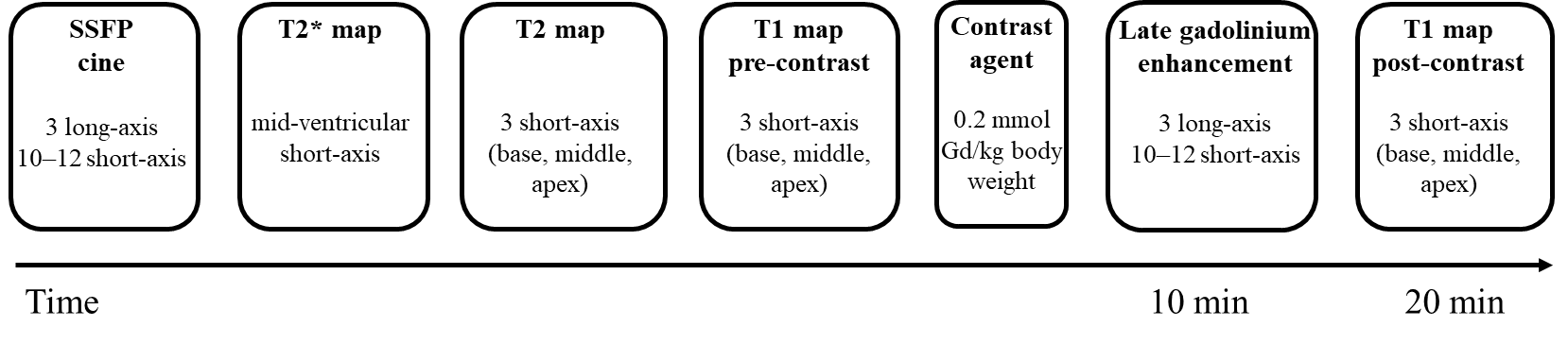


| **Table S1. Clinical Characteristics of the Study Population** | | | | |
| --- | --- | --- | --- | --- |
|  | **HIV-1+/LVEF+ (n=12)** | **HIV-1+/LVEF- (n=35)** | **Normal Controls (n=21)** |  |
|  |  |  |  | **P value** |
| **Variable** |  |  |  |  |
| *Demographics and clinical characteristics* |  |  |  |  |
| Age (y) | 34.8±8.3 | 39.4±8.8 | 40.4±10.9 | 0.239 |
| Men | 11 (91.7)^a^ | 34 (97.1) ^a^ | 12 (57.1) | <0.001* |
| Hypertension | 3 (25.0) ^a^ | 6 (17.1) ^a^ | 0 (0.0) | <0.001* |
| Heart rate (bpm) | 68±3 | 67±10 | 68±9 | 0.183 |
| DM | 2 (16.7) | 5 (14.3) | 0 (0.0) | 0.128 |
| Smoking history | 4 (33.3) | 6 (17.1) | 5 (23.8) | 0.500 |
| Hemoglobin (g/L) | 118.1±8.2 | 122.5±3.1 | 129.2±5.14 | 0.091 |
| Anemia | 3 (25) | 6 (17.1) | NA | 0.864 |
| HIV acquisition risk |  |  |  |  |
| IVDU | 1 (8.3) | 2 (5.7) | NA | 0.818 |
| MSM | 9 (75.0) | 30 (85.7) | NA | 0.684 |
| Heterosexual | 2 (16.7) | 2 (5.7) | NA | 0.566 |
| Blood transfusion recipients | 0 (0.0) | 1 (2.9) | NA | 0.566 |
| Known duration of HIV diagnosis (y) | 4 (2, 8) | 5 (2, 10) | NA | 0.271 |
| AIDS stage | 5 (41.7) | 12 (34.3) | NA | 0.733 |
| ART use |  |  |  |  |
| Current | 9 (75.0) | 30 (85.7) | NA | 0.684 |
| Duration (y) | 2 (1, 4) | 2 (1, 5) | NA | 0.405 |
| *Anthropometric measurements* |  |  |  |  |
| Weight (kg) | 66 (62, 77) | 70 (65, 78) | 65 (60,80) | 0.473 |
| Body mass index (kg/m²) | 23.29 (21.1, 24.3) | 21.21 (19.9, 24.2) | 23.13 (20.8, 26.8) | 0.360 |
| *Laboratory results* |  |  |  |  |
| Current CD4 (cells/mm³) | 578.9±313.3 | 507.7±333.3 | NA | 0.520 |
| Current CD4+/CD8+ ratio (%) | 0.6 (0.2, 0.9) | 0.5 (0.2, 0.7) | NA | 0.373 |
| Plasma HIV RNA<75 copies/ml | 5 (41.7) | 6 (17.1) | NA | 0.181 |
| Creatine (umol/L) | 70.3±10.8 | 66.8±14.7 | NA | 0.447 |
| Glucose (mmol/L） | 5.21 (4.3, 5.3) | 5.21 (4.8, 5.8) | NA | 0.407 |
| Triglyceride level (mg/dL） | 2.32 (1.1, 2.9) | 1.75 (1.2, 2.7) | NA | 0.267 |
| SF (ng/mL) | 189（15.8, 534.5） | 247 (38.1, 394.9) | NA | 0.265 |
| Cholesterol level (mmol/L) |  |  |  |  |
| Total | 4.28 (3.7, 4.8) | 4.32 (3.3, 5.5) | NA | 0.751 |
| HDL | 0.82 (0.8, 1.1) | 093 (0.8, 1.1) | NA | 0.518 |
| LDL | 2.3±0.6 | 2.6±1.3 | NA | 0.410 |

Data are summarized by the mean ± SD if they were abnormally distributed or median and interquartile range if they were abnormally distributed. An n (%) was used for categorical variables. P-values were obtained using one-way ANOVA, the Student t test, Kruskal-Wallis test (for non-normal data), c² test, or Fisher’s exact test. NA = not applicable, * indicates significant difference. a P<0.05 versus normal control subjects. Acronyms: AIDS acquired immunodeficiency syndrome; IVDU intravenous drug users; ART antiretroviral therapy; MSM men who have sex with men; ART antiretroviral therapy; SF Serum ferritin; HDL high-density lipoprotein; LDL low-density lipoprotein.
